# Altered miRNA and mRNA Expression in Sika Deer Skeletal Muscle with Age

**DOI:** 10.3390/genes11020172

**Published:** 2020-02-06

**Authors:** Boyin Jia, Yuan Liu, Qining Li, Jiali Zhang, Chenxia Ge, Guiwu Wang, Guang Chen, Dongdong Liu, Fuhe Yang

**Affiliations:** 1 College of Animal Science and Technology, Jilin Agricultural University, 2888 Xincheng Street, Changchun 130118, China; jiaboyin@jlau.edu.cn (B.J.); liu18104604092@163.com (Y.L.); lqn1005257690@163.com (Q.L.); Zhangjialijlau@163.com (J.Z.); 2 College of Vocational and Technical Education, Changchun Sci-Tech University, 1699 Donghua Street, Changchun 130606, China; gechenxia@126.com; 3 Institute of Wild Economic Animals and Plants and State Key Laboratory for Molecular Biology of Special Economical Animals, Chinese Academy of Agricultural Sciences, 4899 Juye Street, Changchun,130112, China; wangguiwu@caas.cn; 4 Key Laboratory of Straw Biology and Utilization, The Ministry of Education, Jilin Agricultural University, 2888 Xincheng Street, Changchun 130118, China; chg61@163.com; 5 College of Engineering and Technology, Jilin Agricultural University, 2888 Xincheng Street, Changchun 130118, China

**Keywords:** sika deer, microRNA, mRNA, skeletal muscles, age-dependent

## Abstract

Studies of the gene and miRNA expression profiles associated with the postnatal late growth, development, and aging of skeletal muscle are lacking in sika deer. To understand the molecular mechanisms of the growth and development of sika deer skeletal muscle, we used de novo RNA sequencing (RNA-seq) and microRNA sequencing (miRNA-seq) analyses to determine the differentially expressed (DE) unigenes and miRNAs from skeletal muscle tissues at 1, 3, 5, and 10 years in sika deer. A total of 51,716 unigenes, 171 known miRNAs, and 60 novel miRNAs were identified based on four mRNA and small RNA libraries. A total of 2,044 unigenes and 11 miRNAs were differentially expressed between adolescence and juvenile sika deer, 1,946 unigenes and 4 miRNAs were differentially expressed between adult and adolescent sika deer, and 2,209 unigenes and 1 miRNAs were differentially expressed between aged and adult sika deer. Gene Ontology (GO) and Kyoto Encyclopedia of Genes and Genomes (KEGG) analyses showed that DE unigenes and miRNA were mainly related to energy and substance metabolism, processes that are closely associate with the growth, development, and aging of skeletal muscle. We also constructed mRNA–mRNA and miRNA–mRNA interaction networks related to the growth, development, and aging of skeletal muscle. The results show that mRNA (Myh1, Myh2, Myh7, ACTN3, etc.) and miRNAs (miR-133a, miR-133c, miR-192, miR-151-3p, etc.) may play important roles in muscle growth and development, and mRNA (WWP1, DEK, UCP3, FUS, etc.) and miRNAs (miR-17-5p, miR-378b, miR-199a-5p, miR-7, etc.) may have key roles in muscle aging. In this study, we determined the dynamic miRNA and unigenes transcriptome in muscle tissue for the first time in sika deer. The age-dependent miRNAs and unigenes identified will offer insights into the molecular mechanism underlying muscle development, growth, and maintenance and will also provide valuable information for sika deer genetic breeding.

## 1. Introduction

Since the beginning of the 21st century, the popularity of deer farming has been increasing. In many areas, deer farming has become an alternative to traditional agriculture. Different countries have different purposes for deer farming. Asian countries such as China, Japan, and Korea preferred velvet antlers, but western countries preferred venison [1]. For example, New Zealand’s annual sales of venison were more than six times higher than velvet antlers [2]. Venison has the characteristics of high protein, low fat, digestibility, rich nutrition, delicious taste, and can even reduce the risk of cancer [3]. This high-quality venison meets the expectations of modern consumers for a healthy diet. However, the molecular mechanism of muscle growth and development in sika deer was unclear.

Muscle growth was not only crucial for providing meat for humans but was also central to the quality of human life through its involvement in several diseases [4]. Age-related changes in the composition of skeletal muscle were associated with functional limitation, disability, and metabolic disorders [5]. Changes in muscle damage and repair during aging can have serious consequences, leading to muscle inflammation and degeneration [6]. In order to better understand muscle growth, diseases, and gene expression patterns in thr muscle development of different age groups should be characterized in sika deer.

MicroRNAs (miRNAs) are endogenous, evolutionarily conserved, small non-coding RNAs. They are important in the epigenetic regulation of various biological processes of gene expression [7]. MiRNAs regulated gene expression by binding to the 3’ untranslated region (3’UTR) of target mRNAs, resulting in the cleavage or translational inhibition of mRNAs [8]. Therefore, identifying the target of microRNAs was essential to understanding their biological function. The growth and development of skeletal muscle was a complex process, in which miRNA played a crucial regulatory role. Muscle-specific miRNAs included miR-1, miR-133a/b, and miR-206, which were involved in myoblast proliferation and differentiation by regulating the expression of target genes, such as histone deacetylase 4, serum response factor, the myogenic differentiation family, and other myogenic transcriptional factors [9,10,11]. Other non-specific muscle miRNAs have also been involved in the regulation of muscle growth and development, such as miR-17, miR-19, miR-15b, miR-322, miR-208b, miR-26a, miR-30, miR-128a, miR-203a, miR-214, and miR-3906 [12,13,14,15]. For example, miR-26a promoted myoblast differentiation by regulating the expression of target genes Smad1 and Smad4 [16]. Both miR-3906 and miR-203a impaired fast muscle differentiation by targeting Homer-1b or Dmrt2a, respectively [17,18]. Skeletal muscle degenerated progressively, losing mass over time, also known as sarcopenia, which leads to reduced physical ability. During the aging process of skeletal muscle, miRNAs were also involved in regulation, such as Let-7, miR-29, miR-99, miR-100, miR-451, miRNA-144, miRNA-18a, mRNA-15a, and miRNA-181 [19,20,21]. For example, miRNA Let-7 family members Let-7b and Let-7e were increased significantly in the skeletal muscles of the elderly, which are involved in regulating the cell cycle (such as cellular proliferation and differentiation) of let-7 target genes [22]. Aging also altered the up-regulated expression of miR-451, miR-144, miR-18a, and miR-15a and the down-regulated expression of miR-181a/b in skeletal muscle from old rhesus monkeys [5]. Thus, changes in the expression of interrelated miRNAs and target genes play a decisive role in controlling age-related alterations in muscle mass and function. In recent years, Illumina sequencing technology has been increasingly used to identify potential key interactions between miRNAs and mRNAs in the development of skeletal muscle [23,24,25]. However, miRNA-mRNA regulatory networks associated with the muscle growth, development and aging of sika deer have not been studied. 

In the current study, we integrated the miRNA–mRNA paired expression profiling of muscle to identify miRNAs and genes that were differentially expressed among different developmental stages in sika deer. The age-associated miRNA–mRNA regulatory network related to the muscle growth, development, and aging of sika deer was successfully constructed. The analysis of age-dependent miRNA–mRNA interactions throughout the life cycle of muscle development, growth, and maintenance provided new insights into the potential molecular mechanisms and pathways of sika deer genetic breeding.

## 2. Materials and Methods

### 2.1. Animals and Tissues Collection

In the present study, all the sika deer were conducted according to the guidelines established by Jilin Agricultural University. One-year-old (juvenile, Msc_1), three-year-old (adolescence, Msc_2), five-year-old (adult, Msc_3), and ten-year-old (aged, Msc_4) sika deer (three sika deer for each group) were fasted and the following day humanely slaughtered, and the longissimus dorsi muscles were collected. Fresh samples were snap-frozen in liquid nitrogen and then stored at −80 °C until RNA extraction. The processing of all the Sika Deer in the present study strictly followed the approved guidelines of Jilin Agricultural University of the ethics committee (20170321).

### 2.2. RNA Sequencing and Data Analysis

Total RNA was isolated from skeletal muscle samples using TRIzol reagent (Invitrogen, CA, USA). The quality, concentration, and integrity of RNA were checked using a nanodrop photometer and an agilent 2100 bioanalyzer. A total of 4 cDNA libraries were sequenced from the skeletal muscle of juvenile (Msc_1), adolescence (Msc_2), adult (Msc_3), and aged (Msc_4) groups (1 pool of n= 3 for each group). Illumina HiSeq2500 sequencing was carried out, which performs a high-throughput deep sequencing of the sika deer transcriptome. Construction of an mRNA library and sequencing were performed as we have previously described [26]. Prior to differential gene expression analysis, for each sequenced library, the read counts were adjusted by the edge *R* program package through one scaling normalized factor. A differential expression analysis of two samples was performed using the DEGseq (2010) R package. The p value was adjusted using q value [27]. *q* value < 0.005 and |log2(fold change)|>1 was set as the threshold for significantly differential expression.

### 2.3. Small RNA Sequencing and Data Analysis

Equivalent concentrations (1.5 μg) of the RNA of three individuals from skeletal muscle were pooled to construct RNA libraries using a TruSeq small RNA Sample Pre Kit for each developmental stage (Illumina, San Diego, CA, USA). A total of 4 RNA libraries were sequenced from the skeletal muscle of juvenile (Msc_1), adolescence (Msc_2), adult (Msc_3), and aged (Msc_4) groups (1 pool of *n* = 3 for each group). Briefly, fragments 16~35 nt in length were excised and purified from a PAGE gel, and adaptors were ligated to the 5’and 3’ends by T4 RNA ligase. After amplification by RT-PCR, the 140~160 bp PCR products were purified on an 8% polyacrylamide gel (100V, 80 min). The purified cDNA fragments preparations were sequenced on an Illumina Hiseq 2500 platform, and 50 bp single-end reads were generated after removing ploy-N, 3’and 5’adaptor contamination, containing ploy A or T or G or C, and fragments of less than 18 nt from raw data. The clean reads were compared to the Bos taurus reference sequence by Bowtie to annotate all known rRNA, tRNA, scRNA, snRNA, and snoRNA small RNA sequences. DE miRNAs were identified with qvalue<0.01 and |log2 (fold change)|>1 as the threshold.

### 2.4. miRNA–mRNA Interaction Network Construction

Predicting the target unigene of DE miRNA was performed by miRanda. These target unigenes were then compared to transcriptome data. The Pearson correlation coefficients between DE-miRNAs and DE-mRNAs were further calculated. Only when the expression pattern of the target unigene was contrary to its corresponding miRNA can it be used as a candidate target unigene for differentially expressed (DE) miRNA. Finally, the miRNA–mRNA interaction networks were draw by the Cytoscape 3.1.0 (http://www.cytoscape.org/).

### 2.5. Gene Ontology (GO) and Kyoto Encyclopedia of Genes and Genomes (KEGG) Analyses

To further understand the biological and metabolic pathways of the DE unigenes and the miRNA target unigenes, GO analysis (http://geneontology.org) and KEGG analysis (www.genome.jp/kegg) were performed with the DAVID Bioinformatics Resources v6.7 (http://david.abcc.ncifcrf.gov/). A GO biology process and KEGG pathway analysis were conducted based on the whole Bos taurus annotation as the background gene set. Fisher’s exact test was used to define significant GO and KEGG as having a *p*-value less than 0.05.

### 2.6. Real-Time qPCR Validation of Differentially Expressed Unigenes and miRNAs

Total RNA was isolated from skeletal muscle samples by using TRIzol reagent (Invitrogen, Carlsbad, CA, USA). Then, cDNA was synthesized by a reverse-transcription Kit (Takara, Kyoto, Japan). Real-time qPCR reactions were performed using SYBR Premix Ex Taq^TM^ II (Takara, Japan) and were detected in the ABI Prism 7900 Sequence Detection System (Ambion, Austin, TX, USA). GAPDH was used as an endogenous control for mRNA, and U6 snRNA was used as an exogenous control for miRNA. Each reaction was run in triplicate. All experimental data were analyzed using the 2^−∆∆CT^ method.

### 2.7. Supporting Data Information

The raw transcriptome and miRNA data from this study have been submitted to the NCBI Gene Expression Omnibus (https://www.ncbi.nlm.nih.gov/geo/query/acc.cgi?acc=GSE142980).

## 3. Results

### 3.1. Overview of the Transcriptome and the miRNAome

We obtained a total of 26,956,235, 27,853,896, 26,945,504, 26,562,256 raw reads were generated from Msc_1, Msc_2, Msc_3, and Msc_4 groups, respectively, by mRNA sequencing. After removing adaptors, contamination, and low-quality reads, 25,871,143, 26,752,746, 26,674,673, 26,137,950 clean reads were obtained (Table S1). A total of 37,262, 37,192, 34,803, 36,565 unigenes, which FPKM > 0.3, were obtained (Table S2). A total of 23,083 of these unigenes were co-expressed in four development stage. In our previous studies, this data was used as part of the whole body of sika deer transcriptome analysis but did not submit raw data of skeletal muscle or conduct an in-depth analysis [26].

To decipher the characteristics of miRNAs in skeletal muscle among different developmental stages in sika deer, a total of 14,103,998, 10,521,312, 11,544,954, 11,138,451 reads were obtained from the four developmental stages, respectively, by small RNA sequencing. After quality filtering and the removal of contaminant and adaptor reads, 13,828,764 (98.05%), 10,369,882 (98.56%), 11,356,719 (98.37%), 10,910,592 (97.95%) clean reads were obtained, respectively (Table S3). Since the genome of the sika deer has not been sufficiently characterized, we can only refer to the bovine genome database. About 200,240, 168,806, 115,410, 128,106 clean reads were unique small RNA mapping of the bovine reference genome (Table S4). After removing rRNAs, tRNAs, snRNAs, snoRNAs, and other small RNAs that were matched, a total of 928, 808, 743, 855 unique small RNAs were obtained (Table S5). The length distribution of small RNA reads were between 20–24 nt, and read counts with 22 nt were the highest (Figure S1). A total of 171 known miRNAs were identified, including 142, 137, 122 and 138, that were expressed, respectively, in the four developmental stages of sika deer, a nd a total of 60 novel miRNAs were predicted (Table S6). 

### 3.2. DE Unigenes, GO and KEGG Analysis

**Figure 1:**
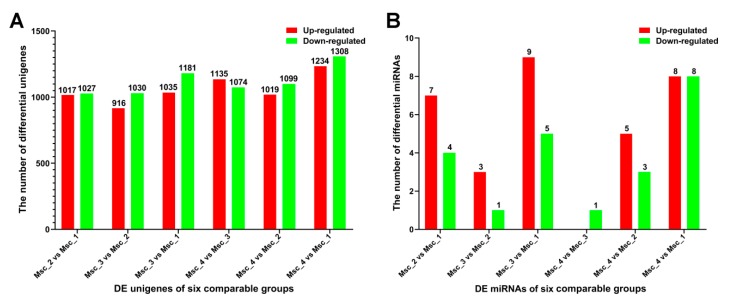
Statistics for DE unigenes and miRNAs in each comparable group. (**A**) Statistics of DE unigenes. (**B**) Statistics of DE miRNAs. *q* value < 0.005 and |log2(fold change)|>1 were used as thresholds of significance for DE unigenes. *q* value < 0.01 and |log2 (fold change)|>1 were used as thresholds of significance for DE miRNAs.

The molecular mechanisms and pathways regulating the skeletal muscle growth, development, and aging of sika deer were related to the age-dependent regulation of abundance of unigenes. In our study, a total of 51716 unigenes were detected in the skeletal muscle of Msc_1, Msc_2, Msc_3, and Msc_4 groups (FPKM > 0.3). Of the 4921 DE unigenes, 1017 up-regulated and 1027 down-regulated unigenes in the Msc_2 vs. Msc_1 group, 916 up-regulated and 1030 down-regulated unigenes in the Msc_3 vs. Msc_2 group, 1035 up-regulated and 1181 down-regulated unigenes in the Msc_3 vs. Msc_1 group, 1135 up-regulated and 1074 down-regulated unigenes in the Msc_4 vs. Msc_3 group, 1019 up-regulated and 1099 down-regulated unigenes in the Msc_4 vs. Msc_2 group, and 1234 up-regulated and 1308 down-regulated unigenes in the Msc_4 vs. Msc_1 group with a criteria of at least a 2-fold change and a *q* value less than 0.005 (Figure 1A, Table S7).

GO biological processes were analyzed using the DAVID Gene Ontology database (*P* < 0.05, Table S8). In the Msc_2 vs. Msc_1 group, the DE unigenes were mainly involved in cell movement, such as cell migration, the regulation of cell adhesion, the regulation of cell migration, the regulation of cellular component movement, the regulation of cell motility, and cell adhesion. In the Msc_3 vs. Msc_2 group and the Msc_4 vs. Msc_3 group, the DE unigenes were mainly related to energy and substance metabolism, such as the ATP metabolic process, the purine nucleoside triphosphate metabolic process, the ribonucleoside triphosphate metabolic process, the purine ribonucleoside triphosphate metabolic process, and the nucleoside triphosphate metabolic process (Table S8). Figure S2 shows the top 20 GO categories in each group. By contrast, the KEGG pathway enrichment did not show the significant differences between these comparisons. These pathways were mainly related to energy and substance metabolism, such as Oxidative phosphorylation, Pyruvate metabolism, Glycolysis/Gluconeogenesis, and so on (Table S9). Figure S3 shows the top 20 enriched KEGG pathways in each group.

### 3.3. DE Unigenes Regulatory Network Analysis

**Figure 2:**
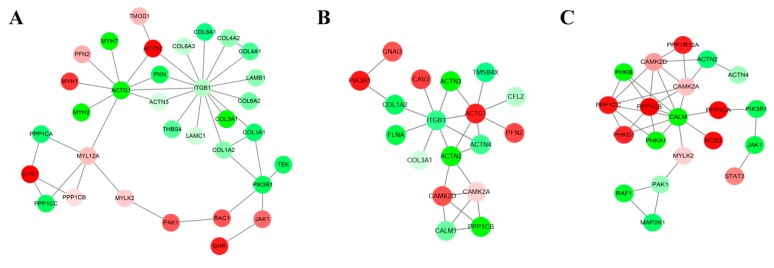
Interaction networks of the top DE unigenes in each comparable group. (**A**) Msc_2 vs. Msc_1. (**B**) Msc_3 vs. Msc_2. (C) Msc_4 vs. Msc_3. The up-regulated unigenes were displayed as red circles, and the down-regulated unigenes were displayed as green circles.

By constructing networks of DE unigenes, 302 (145 up-regulated and 157 down-regulated), 279 (137 up-regulated and 142 down-regulated), and 297 (149 up-regulated and 148 down-regulated) mRNA–mRNA pairs were identified in the Msc_2 vs. Msc_1, Msc_3 vs. Msc_2, Msc_4 vs. Msc_3 comparisons, respectively (Table S10). To identify the functions of networks of DE unigenes and their potential network connections, we used DAVID to determine which unigene networks were enriched in. For the Msc_2 vs. Msc_1 comparison, the top gene–gene interaction network was found to be involved in the PI3K-Akt signaling pathway, the regulation of actin cytoskeleton, and the actin binding (Figure 2A, Table 1). Amongst them, the actin binding was one of the important pathways in this network. A total of 7 target genes of DE unigenes were members of this network, including PFN2, MYH1, MYH2, MYH7, ACTN2, ACTN3, and TMOD1 (Table 1). For the Msc_3 vs. Msc_2 contrast, the top gene network was found to be involved in the regulation of actin cytoskeleton, the cAMP signaling pathway, and focal adhesion. The analysis showed significant differences belonging to regulation of actin cytoskeleton pathway in both the Msc_2 vs. Msc_1 group and the Msc_3 vs. Msc_2 group. The ITGB1 and ACTG1 was the core of this network of DE unigenes (Figure 2B, Table 1). Finally, for the Msc_4 vs. Msc_3 comparison, the top gene network was found to be involved in viral carcinogenesis, the cAMP signaling pathway, the calcium signaling pathway (Figure 2C, Table 1), and especially the calcium signaling pathway, including CALM, PHKG1, PHKB, PHKA1, CAMK2D, MYLK2, NOS3, PPP3CA, and CAMK2A. The CALM was the core of this network of 20 DE unigenes (Table 1).

### 3.4. DE miRNAs, GO, and KEGG Analysis

Statistical analysis of the expression levels of known miRNAs over the lifespan of muscle development was performed. Among the 171 miRNAs, 105 miRNAs were co-expressed in four development stages. A total of 17 miRNAs were expressed only in three development stages, and 19 miRNAs were expressed only in two development stages. In addition, 11, 6, 3, and 10 miRNAs were expressed specifically in the juvenile, adolescent, adult, and aged stage, respectively (Table S11). We also found many differences in the abundances of the different miRNAs. The top 20 most abundant miRNAs of all the development stages were as follows: bta-miR-1, bta-miR-378, bta-miR-133a, bta-miR-26a, bta-miR-26c, bta-miR-378c, bta-miR-199a-3p, bta-miR-3604, bta-miR-486, bta-let-7b, bta-miR-3596, bta-miR-30c, bta-miR-199a-5p, bta-miR-145, bta-miR-133b, bta-miR-7, bta-miR-2898, bta-miR-22-3p, bta-miR-3600, and bta-let-7c (Table S11).

**Figure 3:**
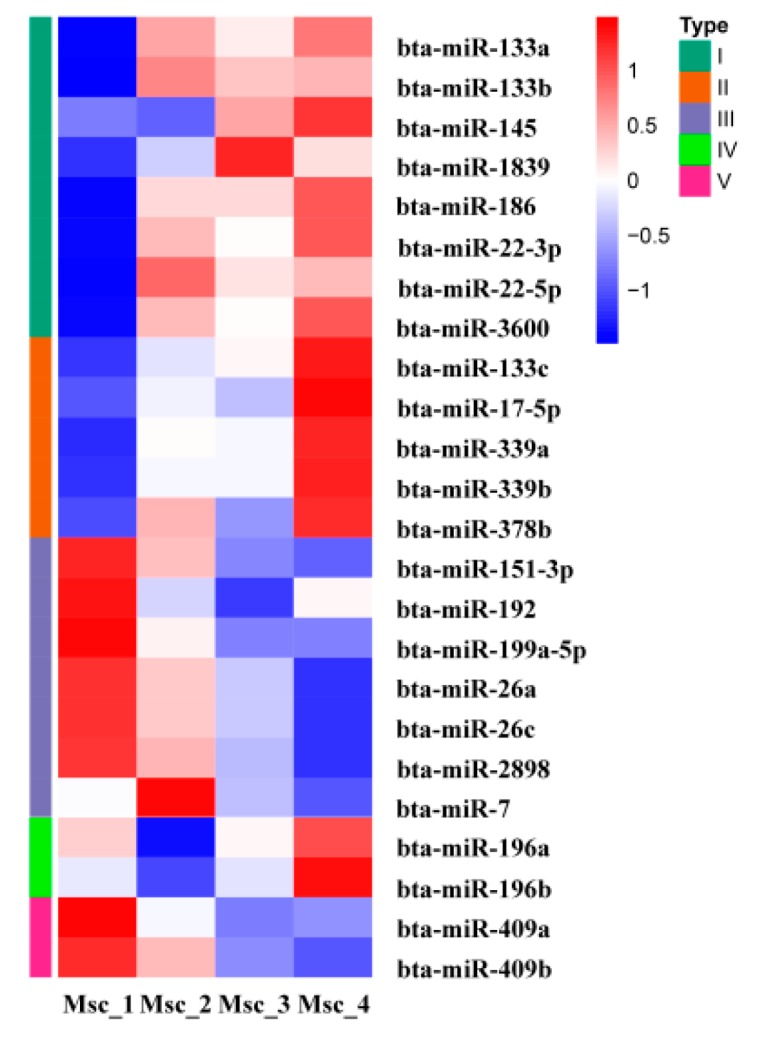
Hierarchical clustering of DE miRNAs.

**Figure 4:**
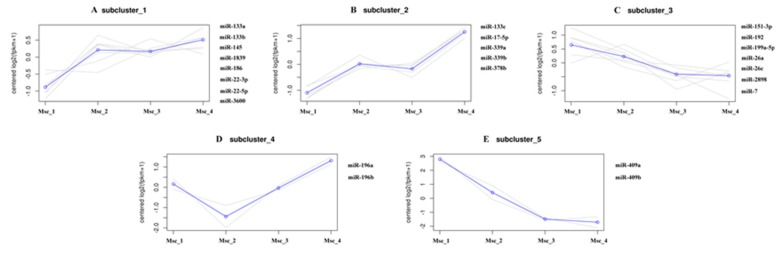
K-means clustering of ED miRNAs. (**A**,**B**,**D**): The expression levels of miRNAs increased gradually after adolescence. (**C**,**E**): The expression levels of miRNAs decreased gradually after adolescence.

To generate DE miRNAs during sika deer muscle development, we detected the level of miRNAs expression patterns at the juvenile, adolescent, adult, and aged stages (Table S12). There were 7 up-regulated and 4 down-regulated miRNAs in the Msc_2 vs. Msc_1 group, 3 up-regulated and 1 down-regulated miRNAs in the Msc_3 vs. Msc_2 group, 9 up-regulated and 5 down-regulated miRNA in the Msc_3 vs. Msc_1 group, 0 up-regulated and 1 down-regulated miRNA in the Msc_4 vs. Msc_3 group, 5 up-regulated and 3 down-regulated miRNA in the Msc_4 vs. Msc_2 group, and 8 up-regulated and 8 down-regulated miRNA in the Msc_4 vs. Msc_1 group (Figure 1B). A hierarchical clustering of each group of DE miRNAs was performed (Figure 3). Meanwhile, a K-means clustering of DE miRNAs showed that the 24 DE miRNAs were divided into five subclusters based on their expression patterns (Figure 4). The expression levels of miRNAs such as miR-133a, miR-133b, miR-145, miR-22-3p, miR-22-5p (Figure 4A), miR-133c, miR-17-5p (Figure 4B), and miR-196a, miR-196b (Figure 4D) increased gradually after adolescence. By contrast, the expression levels of other miRNAs such as miR-151-3p, miR-192, miR-199a-5p, miR-26a, miR-7 (Figure 4C), miR-409a, miR-409b (Figure 4E) decreased significantly. The results suggest that different expression patterns of miRNAs may be closely related to the growth and aging of skeletal muscle.

In total, 11, 4, and 1 miRNAs and 212, 120, and 91 target mRNAs were found form the Msc_2 vs. Msc_1, Msc_3 vs. Msc_2, Msc_4 vs. Msc_3 comparisons, respectively. To further investigate the functional clusters to which these DE miRNAs belonged, GO and KEGG analyses were performed for their target genes. The GO analysis showed that the top 20 GO terms of biological process, cellular component, and molecular function enriched the combinations of Msc_2 vs. Msc_1, Msc_3 vs. Msc_2, and Msc_4 vs. Msc_3, and we found that the only one significant GO in Msc_3 vs. Msc_2 (oxidoreductase activity). The biological process categories were mainly in the metabolic process, substance synthesis, energy synthesis in Msc_2 vs. Msc_1 and Msc_4 vs. Msc_3. Only in Msc_4 vs. Msc_3 did the significant cellular component categories corresponding to miRNAs include actin cytoskeleton, an intermediate filament cytoskeleton, and an extrinsic component of membrane (Figure S4). By contrast, the KEGG pathway enrichment did not show the significant differences among these comparisons. These pathways were mainly related to substance metabolism and nutrient accumulation, such as Glycolysis/Gluconeogenesis, Citrate cycle (TCA cycle), Pyruvate metabolism, and so on (Figure S5). Interestingly, each group of comparisons are all connected to the factors affecting the regulation of blood glucose, such as the Insulin signaling pathway, Insulin resistance, the Glucagon signaling pathway, Type II diabetes mellitus, and Type I diabetes mellitus.

### 3.5. miRNA–mRNA Interaction Analysis

**Figure 5:**
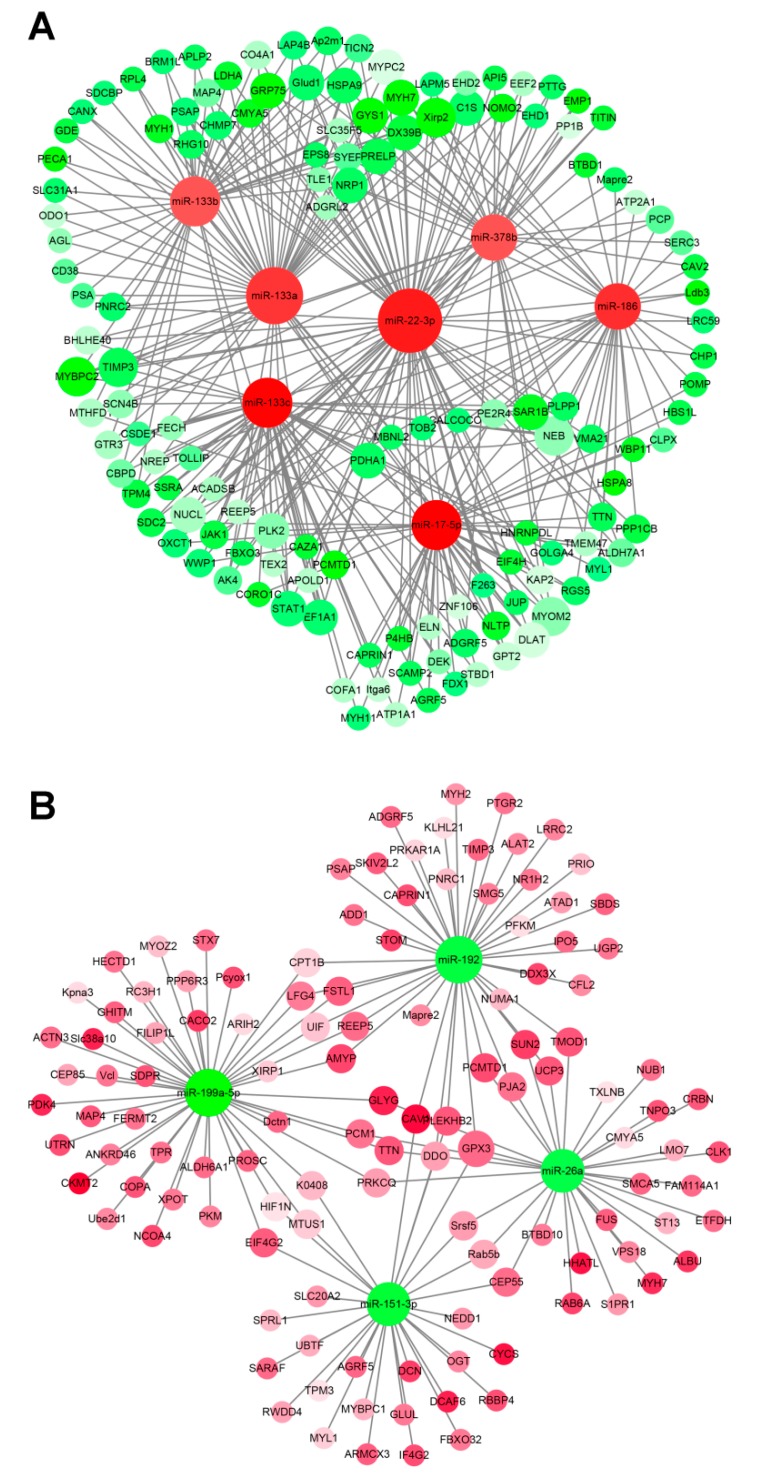
miRNA–mRNA interaction network associated with muscle development in the sika deer. (**A**) Up-regulated miRNAs and down-regulated target genes related to muscle growth and development. (**B**) Down-regulated miRNAs and up-regulated target genes related to muscle growth and development. The up-regulated miRNAs or genes were displayed as red circles, and the down-regulated miRNAs or genes were displayed as green circles.

**Figure 6:**
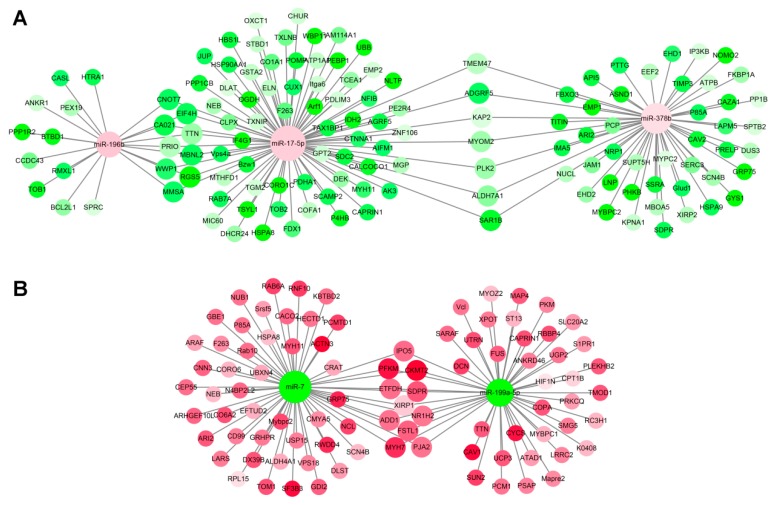
miRNA–mRNA interaction network associated with muscle aging in the sika deer. (**A**) Up-regulated miRNAs and down-regulated target genes related to muscle aging. (**B**) Down-regulated miRNAs and up-regulated target genes related to muscle aging. The up-regulated miRNAs or genes were displayed as red circle, and the down-regulated miRNAs or genes were displayed as green circles.

The regulation process needs to be further studied in order to use miRNAs as new targets for elucidating the mechanisms over the lifespan of muscle development, growth, and maintenance in sika deer. MiRNAs negatively regulate targeted gene expression at the post-transcriptional level by translational repression or by silencing its targeted genes. DE unigenes and DE miRNAs between the different development stages were selected for target prediction and correlation analysis. We focused on the 13 miRNAs related closely to muscle development and aging (Table 2). The results show that 502 miRNA–mRNA pairs associated with muscle development and 253 miRNA–mRNA pairs associated with muscle aging had negative relationships (Figure 5, Figure 6). During muscle development, miRNAs such as miR-133c, miR-22-3p, miR-17-5p, miR-378b, miR-133a, miR-133b, and miR-186 were at the core of the miRNA–mRNA network and were up-regulated, which related to muscle development via more than two potential target genes (Figure 5A). Among them, the important hub genes Myh1, Myh7, Myl1, and DEK, which related to muscle development, were targeted by miR-133a and miR-133c miRNAs. On the contrary, miR-192, miR-199a-5p, and miR-151-3p were down-regulated and targeted Myh2, CFL2, ACTN3, DCN, TPM3, and OGT related to muscle development (Figure 5B). Similarly, during muscle aging, miRNAs such as miR-17-5p and miR-378b were up-regulated and targeted WWP1, DEK, DHCR24, PTTG, and HSPA9 related to muscle aging (Figure 6A). On the contrary, miRNAs such as miR-199a-5p and miR-7 were down-regulated and targeted UCP3, FUS, CAV1, RAB6A, and TOM1 related to muscle aging (Figure 6B). The results show that these miRNAs and their potential target genes may have important roles in muscle development and aging in sika deer.

### 3.6. Validation of mRNA-seq and miRNA-seq Data

**Figure 7:**
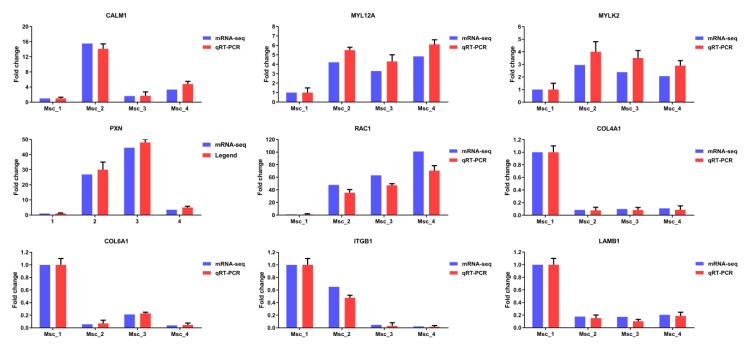
qRT-PCR validation of mRNA-seq data. The expression levels of nine differentially expressed genes (CALM1, COL4A1, COL6A1, ITGB1, LAMB1, RAC1, MYL12A, MYLK2, and PXN) were normalized against GAPDH. The vertical axis indicates the normalized genes expression level for each stage. For each gene, the expression level and sequencing abundance in Msc_1 were given as a negative control and set at 1.

**Figure 8:**
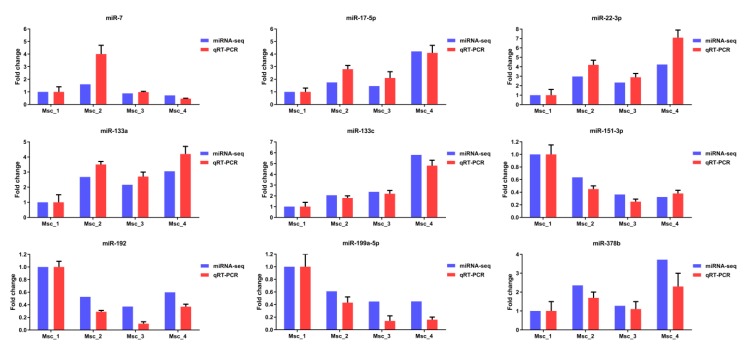
qRT-PCR validation of miRNA-seq data. The expression levels of nine differentially expressed miRNAs (miR-133a, miR-133c, miR-17-5p, miR-22-3p, miR-378b, miR-192, miR-151-3p, miR-199a-5p, and miR-7) were normalized against U6 snRNA. The vertical axis indicates the normalized miRNAs expression level for each stage. For each miRNA, the expression level and sequencing abundance in Msc_1 were given as a negative control and set at 1.

To validate the differential expression of unigenes and miRNA, 9 unigenes and 9 miRNAs were selected for qRT-PCR analysis. As the age increases, the expression of mRNAs (CALM1, COL4A1, COL6A1, ITGB1, LAMB1, RAC1, MYL12A, MYLK2, and PXN) showed significant differences between four groups (Figure 7). Similarly, as the age increases, the expression of miRNAs (miR-133a, miR-133c, miR-17-5p, miR-22-3p, miR-378b, miR-192, miR-151-3p, miR-199a-5p, and miR-7) showed significant differences between four groups (Figure 8). The vertical axis indicates the normalized unigenes and miRNAs expression level for each stage. For each unigene, the expression level and sequencing abundance in Msc_1 were given as a negative control and were set at 1. Therefore, the qRT-PCR analyses largely confirmed the results of mRNA-seq and miRNA-seq data. The expression levels of these mRNAs and miRNAs determined by qRT-PCR were consistent with mRNA-seq and the miRNA-seq data, which validated their accuracy.

## 4. Discussion

In recent years, deep sequencing has been used to identify the interaction between mRNA and miRNA by determining the negative expression correlation between miRNA and target mRNA. In this study, we identified the expression patterns of mRNAs and miRNAs among different developmental stages in sika deer skeletal muscle. We obtained a total of 25,871,143 (juvenile), 26,752,746 (adolescence), 26,674,673 (adult), and 26,137,950 (aged) clean reads from mRNA-seq, and 13,828,764 (juvenile), 10,369,882 (adolescence), 11,356,719 (adult), and 10,910,592 (aged) clean reads from miRNA-seq. This result was very important for the quantitative analysis of unigene and miRNA expression. We also obtained some low-read data, indicating that low-abundance unigenes and miRNAs can also be detected and quantified for subsequent analysis.

We identified 4,921 DE unigenes from the muscles of sika deer in the Msc_2 vs. Msc_1, Msc_3 vs. Msc_2, Msc_4 vs. Msc_3 groups. In the Msc_2 vs. Msc_1 group, the DE unigenes were mainly involved in cell movement. In the Msc_3 vs. Msc_2 group and the Msc_4 vs. Msc_3 group, the DE unigenes were mainly related to energy and substance metabolism. By contrast, the KEGG pathway enrichment did not show the significant differences among these comparisons. These pathways were mainly related to energy and substance metabolism. Therefore, DE unigenes corresponded to three key functions/pathways, which are related to energy metabolism, substance metabolism, and cell movement. These key functions/pathways were probably the most important three key factors to regulate muscle growth and aging.

The DE unigenes network obtained in our study revealed the regulatory relationship of genes after transcription. For the Msc_2 vs. Msc_1 comparison, the top gene–gene interaction network was found to be involved in the PI3K-Akt signaling pathway, the regulation of actin cytoskeleton, and actin binding. Among them, the actin binding signal pathway played an important role in the initial assembly, growth, and maintenance of sarcomeres [28]. In our study, the most significant differential expression of unigenes critical for actin binding, such as MYH1, MYH2, MYH7, and ACTN3, was observed. PI3K-Akt was also an important regulatory pathway for the growth and development of skeletal muscle, for example, it was closely related to the proliferation and differentiation of skeletal muscle, muscle hypertrophy, etc. [29,30]. In addition, the analysis showed significant differences belonging to the regulation of the actin cytoskeleton pathway in both the Msc_2 vs. Msc_1 group and the Msc_3 vs. Msc_2 group. ACTG1, ACTN2, and ACTN3, the most significant differential expression of unigenes critical for the regulation of actin cytoskeleton pathway was observed. Actin and actin-related proteins have been shown to control various cellular processes, including cell volume changes, cell size, and motility [31]. For the Msc_4 vs. Msc_3 comparison, the top gene–gene interaction network was found to be involved in the cAMP signaling pathway, the Calcium signaling pathway, and Viral carcinogenesis. Among them, the cAMP signaling pathway and the Calcium signaling pathway were important regulatory pathways for the aging [32,33]. In CALM, CAMK2A, and CAMK2D, the most significant differential expression of unigenes critical for the cAMP signaling pathway and the Calcium signaling pathway was observed. CALM has been known to play an important role in the aging process by regulating calcium in cells [34]. In summary, through genes regulatory network analysis, we had obtained the core regulatory unigenes that were important for sika deer muscle growth, development, and aging, such as MYH1, MYH2, MYH7, ACTN3, ACTG1, ACTN2, and CALM.

Mammalian skeletal muscle development has been confirmed to be regulated by many miRNAs. However, many of the miRNAs involved are still unknown. In this study, a total of 171 known miRNAs and 60 novel miRNAs and were identified and predicted. So far, there are no such complete reports on deer muscle miRNAs, so our findings will enrich what we know about miRNAs related to muscle growth, development, and aging. The expression level of miRNAs had obvious temporal characteristics. In this study, 105 of 171 miRNAs were co-expressed in four development stages, but these miRNAs were sequenced at variable frequencies from the adolescent to aged stage. The expression level of miRNAs also showed distinct developmental stage specificity. We found that some specific miRNAs were expressed at specific stages. Thus, miRNAs such as miR-23a, miR-199b, miR-381, and so on were expressed specifically in the juvenile stage; miR-1277, miR-1307, miR-2334, and so on were expressed specifically in the adolescent stage; miR-10b, miR-191, and miR-362-3p were expressed specifically in the adult stage; and miR-27a-3p, miR-29b, miR-30d, and so on were expressed specifically in the aged stage. These development stage-specific miRNAs were expressed at very low levels, but some, such as miR-199b, miR-23a, miR-381, and miR-27a-3p, were closely related to growth and development [23,35,36,37]; miR-191 and miR-29b were closely related to aging [38,39]. This result indicates that miRNAs with development-stage specificity might play an important role in the muscle development and aging of sika deer.

In addition, the expression level of some miRNAs was high during the muscle development of sika deer. In this study, miR-1, miR-378, miR-133a, and many miRNAs (e.g., miR-26a, miR-26c, miR-378c, miR-199a-3p, etc.) were the most abundant miRNAs in sika deer skeletal muscle. Several studies have reported transcriptome and miRNAome analyses in the skeletal muscles of ruminants. In a study by Sun et al., miRNA-206, miRNA-1, miRNA-133, miRNA-12, and miRNA-17 were the most abundant miRNAs in the skeletal muscles of bos taurus [40]. In a study by Sheng et al., miR-1, miR-133, miR-181a, and miR-206 were the most abundant miRNAs in the skeletal muscles of ovis aries [41]. This result suggested that there were significant differences in the abundance of miRNA expression in skeletal muscle between different ruminants. Due to the lack of analysis on the expression of miRNAs in different tissues of sika deer, miRNAs specifically expressed in muscle could not be determined in sika deer.

This study provided valuable mRNAs and miRNAs of sika deer muscles for the first time, allowing us to understand the molecular mechanism of sika deer muscle development more comprehensively.

A total of 24 miRNAs were identified to be differentially expressed at different stages of development. For example, the expression level of miRNAs such as miR-133a, miR-133b, miR-186, and miR-22-3p, which were associated with muscle growth and development, decreased gradually. On the contrary, the expression level of miRNAs related to aging, such as miR-199a-5p and miR-7, increased significantly. The result indicates that the miRNAs characterized by temporal or differential expression were closely related to muscle growth, development, and aging.

We identified 16 DE miRNAs and 423 target genes from sika deer muscles in the Msc_2 vs. Msc_1, Msc_3 vs. Msc_2, and Msc_4 vs. Msc_3 groups. In order to investigate how these DE miRNAs regulate muscle growth, development, and aging, through their interaction with target genes, GO and KEGG analyses were performed for the 423 target genes. These target genes corresponded to the key GO functions, which were mainly in the metabolic process, substance synthesis, and energy synthesis in Msc_2 vs. Msc_1. It was speculated that this result was related to the rapid growth of muscle from juveniles to adolescents, which requires a lot of energy and completes the metabolic process. Then, there was no significant difference in Msc_3 vs. Msc_2. This showed little change in muscle growth and aging from adolescence to adulthood. Only in Msc_4 vs. Msc_3, in addition to the metabolism process and energy synthesis, was there also important GO enrichment, such as actin cytoskeleton and intermediate filament cytoskeleton. This result suggested that the aging process of muscle was involved the changes of energy, metabolism, and skeletal muscle. It can be seen that DE miRNAs regulate the growth, development, and aging of muscles by controlling these biological functions of target genes in sika deer. By contrast, the KEGG pathway enrichment did not show the significant differences among these comparisons. These pathways were mainly related to substance metabolism and nutrient accumulation. Therefore, we speculated that DE miRNAs’ regulation of muscle growth and aging in sika deer was the utilization of pathways related to metabolism and nutrient accumulation.

In vivo, miRNAs regulated various biological processes in the network through their target genes. We constructed the miRNA–mRNA network related to muscle growth and development in sika deer. In the network, miR-133c, miR-22-3p, miR-17-5p, miR-378b, miR-133a, miR-133b, and miR-186 were at the core of the miRNA-mRNA network and were up-regulated, which related to muscle development via target genes. The target genes, comprising Myh1, Myh2, Myh7, My11, and DEK, were the key nodes in the network. Among these node genes, the Myosin heavy chain (MyHC) had four isoforms, which were namely Myh1, Myh2, Myh4, and Myh7 [42]. These MyHC isoforms were expressed during the life cycle of skeletal muscle development, which influenced the contraction–relaxation activity in skeletal muscles and were involved in the formation of the cytoskeleton and determining muscle composition [43]. Myh1 was important for the fast-twitch muscle fibers, Myh7 contributed to the slow-twitch muscle fibers, while Myh2 acted as an intermediator between Myh1 and Myh7 [44,45]. Myh1, Myh2, and Myh7 were the signature genes of myoblast differentiation [46]. Our data displayed that the up-regulated expression of miR-133a inhibited the expression of two potential target genes (Myh1 and Myh7) with age. On the contrary, the down-regulated expression of miR-192 promoted the expression Myh2 with age. Therefore, it was speculated that miR-133a and miR-192 were implicated in muscle fiber types, characteristics and myoblast differentiation by affecting the expression of Myh1, Myh2 and Myh7 in sika deer. Another important node gene, Myl1, was an early marker of differentiating fast muscle, and its transcript levels declined with development in post-somitogenic stages [47,48]. The knockdown of Myl1 has been confirmed to cause impairment in chicken myoblast/myotube cultures [49]. It can also lead to significantly reduced motility the and gross impairment of myofibre organization in zebrafish [47]. This suggests that MYL1was critical for muscle function. In our study, the up-regulated expression of miR-133c inhibited the expression of the target gene Myl1 with age. In addition, DEK highly induced satellite cell activation and proliferated the expansion of the transit-amplifying myogenic progenitors [50]. Our results suggest that up-regulation of miR-133c in actively maintaining the quiescent state of an adult stem-cell population by inhibiting the expression of the target gene DEK.

In the network, miR-192, miR-199a-5p, miR-151-3p, and miR-151-3p were at the core of the miRNA–mRNA network and were down-regulated, which related to muscle development via target genes. The target genes, comprising CFL2, ACTN3, DCN, and OGT, were the key nodes in the network. Among these node genes, CFL2, as a member of the cofilin family, was predominantly expressed in skeletal and cardiac muscles. CFL2 localized between Z-discs in myofibrils, which affected cell proliferation, apoptosis, migration, and invasion by regulating actin filaments [51]. In addition, the expression of four MyHC isoforms in undifferentiated myoblast cells with CFL2 RNAi was found to be significantly decreased compared with that in differentiated myoblast cells [52]. Our result indicated that the down-regulated expression of miR-192 may be involved in the regulation of MyHC via the up-regulated expression of CFL2. ACTN3, as the main component of the skeletal muscle Z-discs, was expressed only in fast muscle [53]. As ACTN3 controlled the proportion of fiber types, it was considered one of the main genetic factors determining muscle strength [54]. In addition, ACTN3 function was related to the regulation of muscle structure, muscle contraction, cell metabolism, and calcium pathway [55,56,57]. Our result indicates that the down-regulated expression of miR-199a-5p may be involved in the regulation of muscle strength via the up-regulated expression of ACTN3. DCN promoted muscle fiber hypertrophy by competitively binding to myostatin [58]. DCN could also regulate muscle growth and development by inhibiting the activity of IGF1R and activating AKT [59]. Our result indicates that the down-regulated expression of miR-151-3p may promote the proliferation and differentiation of skeletal muscle cells by up regulating the expression of DCN. A previous study demonstrated that OGT might regulate nutrient-sensitive intracellular processes that mediate cellular metabolism, growth, proliferation, and/or tissue function in skeletal muscle [60,61]. It could be seen that the DE miRNAs mentioned above might regulate the muscle growth and development through their target genes.

In the network, miR-17-5p and miR-378b were at the core of the miRNA–mRNA network and were up-regulated, which related to muscle aging via the target genes. It has been reported that the overexpression of miR-378 significantly reduced the skeletal muscle mass in mice. Mir-378 attenuated muscle regeneration by delaying the activation and differentiation of satellite cells in mice, especially in sarcopenia [62]. However, there were relatively few studies on target genes for miR-378 to regulate myopenia. In this study, the overexpression of miR-378 in the muscle of the aged sika deer was likely to be closely related to myopenia. The target genes, comprising WWP1, DEK, DHCR24, PTTG, and HSPA9, were the key nodes in the network. The reduction of wwp-1 by RNAi completely suppresses the extended longevity of Caenorhabditis elegans, highlighting its important role in aging [63]. Subsequently, it was found that WWP1 mutation could shorten the lifespan of Caenorhabditis elegans through its participation in the insulin/IGF-1 signaling network [64]. Moreover, the overexpression of the WWP1 delayed senescence in human fibroblasts through inhibited the replicative senescence induced by p27Kip1 by promoting p27Kip1 degradation [65]. DEK as proto-oncogene may have senescence inhibitory function [66]. It has been confirmed in the cervical cancer cell that the expression of DEK was down-regulated, which could promote cell aging, while the exogenous overexpression of DEK could prolong cell life [67]. It was later confirmed that DEK can prolong the life of keratinocytes by acting on the p53 pathway [68]. DHCR24, as an enzyme that metabolizes desmosol to cholesterol, was closely related to aging diseases such as Alzheimer’s disease. It has been reported that the activity of the gene coding for the enzyme DHCR24 was selectively reduced in the affected areas of the brain in Alzheimer’s disease [69]. Chesnokova et al. found that PTTG deletion in pituitary cells triggered the p53/p21 aging signal pathway and then induced a DNA damage checkpoint response, which lead to aging and prevented tumor occurrence and malignant transformation [70]. The overexpression of HSPA9 in normal cells could effectively prolong cell life, while the inhibition of HSPA9 expression in tumor cells could lead to senescence or apoptosis of tumor cells [71].

In the network, miR-199a-5p and miR-7 were at the core of the miRNA–mRNA network and were down-regulated, which related to muscle aging via target genes. The target genes, comprising UCP3, FUS, CAV1, NCL, and TOM1, were the key nodes in the network. Sarcopenia was measured through the reduction of hand grip strength. Studies have found that genetic polymorphisms in the UCP3 gene were suggested to be associated with hand grip performances in the elderly population’s skeletal muscle. This suggested that the uncoupling process was related to muscle metabolism/catabolism in the elderly [72,73]. In addition, there was evidence that UCP2, UCP3, and UCP4 affect the individual’s chances of surviving up to a very old age. It was confirmed that the uncoupling pathway as a human candidate pathway was related to longevity [74]. FUS was a prion like protein. Previous studies have shown that FUS plays an important role in age-related diseases, such as neurodegenerative diseases, by forming liquid compartments at sites of DNA damage and in the cytoplasm upon stress in cell [75]. CAV1 expression was significantly increased during aging and was directly bound to Toll-like receptor 5 to induce aging-related signaling and the production of proinflammatory cytokines. Inhibiting the expression of CAV1 could improve the activity of senescent cells, and further exert the effects of anti-oxidative damage and anti-aging [76]. NCL played an important role in cell senescence, mainly through the interaction with TERT to participate in the dynamic localization of telomerase complex in cells [77]. TOM1 was a lysosomal-related gene, which was highly expressed in cell senescence. It was found that the expression of the TOM1 gene can promote the expression of p16 and p21, thus accelerating cell aging. On the contrary, inhibition of the TOM1 gene expression slowed down cell aging [78]. In view of this, the interactive networks mentioned above reflect the complexity of the post-transcriptional regulation of muscle aging in sika deer. A few miRNAs may play a key role in this process, while most miRNAs maintain the stability of the regulatory network.

## 5. Conclusions

In the present study, we comprehensively analyzed the changes in the mRNA and miRNA profiles of skeletal muscle at 1, 3, 5, and 10-years in sika deer, which shows the distinct phenotypes of muscle growth, development, and aging. From these data, we identified unigenes and miRNAs that were differentially expressed among the different development stages. DE unigenes and miRNAs were functionally related to energy and substance metabolism. In addition, we identified key candidate unigenes and miRNAs by using the core functional mRNA–mRNA and miRNA–mRNA network analysis, which were associated with sika deer muscle growth, development, and aging. The integrative miRNA–mRNA analysis of the different development stages of muscles was reported for the first time and suggested a role for miRNA–mRNA interactions in muscle development and molecular breeding.

## Figures and Tables

**Table 1:** Summary of the represented networks generated by pathway analysis.

Data	Molecules in Networks	Score	*P*-Value	Top Functions
Msc_2vs.Msc_1	COL4A2, COL4A1, COL3A1, ITGB1, COL6A3, TEK, RAC1, COL1A2, COL6A2, GYS1, JAK1, COL6A1, COL1A1, LAMC1, LAMB1, PIK3R1, GHR, THBS4	33	0.001	PI3K-Akt signaling pathway
	MYLK2, ACTN2, MYL12A, ACTN3, PPP1CC, ITGB1, PPP1CB, PXN, ACTG1, PPP1CA, PFN2, RAC1, PAK1, PIK3R1		0.016	Regulation of actin cytoskeleton
	PFN2, MYH1, MYH2, MYH7, ACTN2, ACTN3, TMOD1		0.001	actin binding
Msc_3vs.Msc_2	ACTG1, PFN2, ACTN4, CFL2, TMSB4X, ACTN2, ACTN3, ITGB1, PPP1CB, PIK3R1	18	0.034	Regulation of actin cytoskeleton
	GNAI3, CAMK2D, PPP1CB, CAMK2A, PIK3R1, CALM1		0.050	cAMP signaling pathway
	CAV2, ACTN4, COL3A1, ACTN2, ACTN3, ITGB1, PPP1CB, FLNA, ACTG1, COL1A2, PIK3R1		0.005	Focal adhesion
Msc_4vs.Msc_3	ACTN4, ACTN2, STAT3, JAK1, PIK3R1	20	0.005	Viral carcinogenesis
	MAP2K1, RAF1, PPP1CC, PPP1CB, CALM, CAMK2D, PPP1R12A, PAK1, CAMK2A, PIK3R1		0.005	cAMP signaling pathway
	CALM, PHKG1, PHKB, PHKA1, CAMK2D, MYLK2, NOS3, PPP3CA, CAMK2A		0.050	Calcium signaling pathway

**Table 2:** miRNAs identified in the skeletal muscle of sika deer as well as those associated with muscle development and aging in other species.

miRNA ID	Muscle Development	miRNA ID	Muscle Aging
miR-17-5p	skeletal muscle proliferation and differentiation	miR-7	ageing-related inflammation
miR-22-3p	skeletal muscle proliferation and differentiation	miR-17-5p	restored the osteogenic capacity of old mesenchymal stem cells and aging-associated pathologies
miR-26a	skeletal muscle differentiation and regeneration	miR-196b	regulating the muscle protein synthesis pathway
miR-133a	skeletal muscle proliferation, differentiation, and hypertrophy	miR-199a-5p	regulating the MPS pathway
miR-133b		miR-378b	myogenic differentiation of myogenic progenitors from adult and old individual
miR-133c			
miR-151-3p	skeletal muscle proliferation and differentiation		
miR-186	skeletal muscle differentiation		
miR-192	skeletal muscle proliferation and differentiation		
miR-199a-5p	skeletal muscle proliferation and differentiation		
miR-378b	skeletal muscle autophagy and apoptosis		

## Supplementary Materials

Supplementary materials can be found at https://www.mdpi.com/2073-4425/11/2/172/s1. Table S1: Summary of transcriptome data. Table S2: FPKM statistical of muscle transcriptome in sika deer. Table S3: Summary of miRNAome data. Table S4: Statistical information of small RNAs mapping to reference sequence. Table S5: Small RNAs annotation. Table S6: Identification of known miRNAs and novel miRNAs. Table S7: The lists of DE unigenes. Table S8: GO categories of differential unigenes in four developmental stages. Table S9: KEGG pathways of differential unigenes in four developmental stages. Table S10: Interaction networks of DE unigenes in each comparable group. Table S11: Known miRNA four development stages. Table S12: The lists of DE miRNAs. Figure S1: Characterization of miRNA-seq mapped reads from the small RNA sequencing of sika deer muscles. Figure S2: The top 20 GO categories of DE unigenes in each comparable group. Figure S3. The top 20 enriched KEGG pathways of DE unigenes in each comparable group. Figure S4. Top 20 enriched GO terms for the potential genes targeted by DE miRNAs. Figure S5. Top 20 enriched KEGG pathways for the potential genes targeted by DE miRNAs.

## References

[1] Ludwiczak A., Stanisz M., Bykowska M., Skladanowska J., Slosarz P. (2017). Effect of storage on quality traits of the semimembranosus muscle of farmed fallow deer (Dama dama) bucks and does. Anim. Sci. J..

[2] Lin W., Ju G., Zhang A. (2014). Nutritional properties and production situation of venison. J. Econ. Anim..

[3] Triumf E.C., Purchas R.W., Mielnik M., Maehre H.K., Elvevoll E., Slinde E., Egelandsdal B. (2012). Composition and some quality characteristics of the longissimus muscle of reindeer in Norway compared to farmed New Zealand red deer. Meat Sci..

[4] Sun J., Sonstegard T.S., Li C., Huang Y., Li Z., Lan X., Zhang C., Lei C., Zhao X., Chen H. (2015). Altered microRNA expression in bovine skeletal muscle with age. Anim. Genet..

[5] Mercken E.M., Majounie E., Ding J., Guo R., Kim J., Bernier M., Mattison J., Cookson M.R., Gorospe M., de Cabo R. (2013). Age-associated miRNA alterations in skeletal muscle from rhesus monkeys reversed by caloric restriction. Aging (Albany NY).

[6] Goto M. (2008). Inflammaging (inflammation + aging): A driving force for human aging based on an evolutionarily antagonistic pleiotropy theory?. Biosci. Trends.

[7] Carrington J.C., Ambros V. (2003). Role of microRNAs in plant and animal development. Science.

[8] Rajewsky N. (2006). microRNA target predictions in animals. Nat. Genet..

[9] Chen J.F., Tao Y., Li J., Deng Z., Yan Z., Xiao X., Wang D.Z. (2010). Microrna-1 and microRNA-206 regulate skeletal muscle satellite cell proliferation and differentiation by repressing Pax7. J. Cell Biol..

[10] Huang M.B., Xu H., Xie S.J., Zhou H., Qu L.H. (2011). Insulin-like growth factor-1 receptor is regulated by microRNA-133 during skeletal myogenesis. PLoS ONE.

[11] Chen X., Wang K., Chen J., Guo J., Yin Y., Cai X., Guo X., Wang G., Yang R., Zhu L. (2009). In vitro evidence suggests that miR-133a-mediated regulation of uncoupling protein 2 (UCP2) is an indispensable step in myogenic differentiation. J. Biol. Chem..

[12] Kong D., He M., Yang L., Zhou R., Yan Y.Q., Liang Y., Teng C.B. (2019). MiR-17 and miR-19 cooperatively promote skeletal muscle cell differentiation. Cell Mol. Life Sci..

[13] Zhao M.J., Xie J., Shu W.J., Wang H.Y., Bi J., Jiang W., Du H.N. (2019). MiR-15b and miR-322 inhibit SETD3 expression to repress muscle cell differentiation. Cell Death Dis..

[14] Wang J., Song C., Cao X., Li H., Cai H., Ma Y., Huang Y., Lan X., Lei C., Ma Y. (2019). MiR-208b regulates cell cycle and promotes skeletal muscle cell proliferation by targeting CDKN1A. J. Cell Physiol..

[15] Mok G.F., Lozano-Velasco E., Munsterberg A. (2017). MicroRNAs in skeletal muscle development. Semin. Cell Dev. Biol..

[16] Dey B.K., Gagan J., Yan Z., Dutta A. (2012). MiR-26a is required for skeletal muscle differentiation and regeneration in mice. Genes Dev..

[17] Lin C.Y., Chen J.S., Loo M.R., Hsiao C.C., Chang W.Y., Tsai H.J. (2013). MicroRNA-3906 regulates fast muscle differentiation through modulating the target gene homer-1b in zebrafish embryos. PLoS ONE.

[18] Lu C., Wu J., Xiong S., Zhang X., Zhang J., Mei J. (2017). MicroRNA-203a regulates fast muscle differentiation by targeting dmrt2a in zebrafish embryos. Gene.

[19] Hu Z., Klein J.D., Mitch W.E., Zhang L., Martinez I., Wang X.H. (2014). MicroRNA-29 induces cellular senescence in aging muscle through multiple signaling pathways. Aging (Albany NY).

[20] Kim J.Y., Park Y.K., Lee K.P., Lee S.M., Kang T.W., Kim H.J., Dho S.H., Kim S.Y., Kwon K.S. (2014). Genome-wide profiling of the microRNA-mRNA regulatory network in skeletal muscle with aging. Aging (Albany NY).

[21] Mitchell C.J., D’Souza R.F., Schierding W., Zeng N., Ramzan F., O’Sullivan J.M., Poppitt S.D., Cameron-Smith D. (2018). Identification of human skeletal muscle miRNA related to strength by high-throughput sequencing. Physiol. Genom..

[22] Drummond M.J., McCarthy J.J., Sinha M., Spratt H.M., Volpi E., Esser K.A., Rasmussen B.B. (2011). Aging and microRNA expression in human skeletal muscle: A microarray and bioinformatics analysis. Physiol. Genom..

[23] Sun L., Lu S., Bai M., Xiang L., Li J., Jia C., Jiang H. (2019). Integrative microRNA-mRNA Analysis of Muscle Tissues in Qianhua Mutton Merino and Small Tail Han Sheep Reveals Key Roles for oar-miR-655-3p and oar-miR-381-5p. DNA Cell Biol..

[24] Li Z., Abdalla B.A., Zheng M., He X., Cai B., Han P., Ouyang H., Chen B., Nie Q., Zhang X. (2018). Systematic transcriptome-wide analysis of mRNA-miRNA interactions reveals the involvement of miR-142-5p and its target (FOXO3) in skeletal muscle growth in chickens. Mol. Genet. Genom..

[25] Zhang X., Cai S., Chen L., Yuan R., Nie Y., Ding S., Fang Y., Zhu Q., Chen K., Wei H. (2019). Integrated miRNA-mRNA transcriptomic analysis reveals epigenetic-mediated embryonic muscle growth differences between Wuzhishan and Landrace pigs1. J. Anim. Sci..

[26] Jia B.Y., Ba H.X., Wang G.W., Yang Y., Cui X.Z., Peng Y.H., Zheng J.J., Xing X.M., Yang F.H. (2016). Transcriptome analysis of sika deer in China. Mol. Genet. Genom..

[27] Storey J.D., Tibshirani R. (2003). Statistical significance for genomewide studies. Proc. Natl. Acad. Sci. USA.

[28] Ono S. (2014). Regulation of structure and function of sarcomeric actin filaments in striated muscle of the nematode Caenorhabditis elegans. Anat. Rec. (Hoboken).

[29] Wang X., Cao X., Dong D., Shen X., Cheng J., Jiang R., Yang Z., Peng S., Huang Y., Lan X. (2019). Circular RNA TTN Acts As a miR-432 Sponge to Facilitate Proliferation and Differentiation of Myoblasts via the IGF2/PI3K/AKT Signaling Pathway. Mol. Ther. Nucleic Acids.

[30] Zheng L.F., Chen P.J., Xiao W.H. (2019). Signaling pathways controlling skeletal muscle mass. Sheng Li Xue Bao.

[31] Ropka-Molik K., Bereta A., Zukowski K., Piorkowska K., Gurgul A., Zak G. (2017). Transcriptomic gene profiling of porcine muscle tissue depending on histological properties. Anim. Sci. J..

[32] Hamezah H.S., Durani L.W., Yanagisawa D., Ibrahim N.F., Aizat W.M., Bellier J.P., Makpol S., Ngah W.Z.W., Damanhuri H.A., Tooyama I. (2018). Proteome profiling in the hippocampus, medial prefrontal cortex, and striatum of aging rat. Exp. Gerontol..

[33] Kanai S., Hosoya H., Ohta M., Miyasaka K. (2007). Decreased hydrogen-potassium-activated ATPase (H+-K+-ATPase) expression and gastric acid secretory capacity in aged mice. Arch. Gerontol. Geriatr..

[34] Logan S., Cameron J.A., Vig P.J. (2003). Calmodulin activity in aging rat heart. Biomed. Instrum..

[35] Zhu L., Hou L., Ou J., Xu G., Jiang F., Hu C., Wang C. (2019). MiR-199b represses porcine muscle satellite cells proliferation by targeting JAG1. Gene.

[36] Wang L., Chen X., Zheng Y., Li F., Lu Z., Chen C., Liu J., Wang Y., Peng Y., Shen Z. (2012). MiR-23a inhibits myogenic differentiation through down regulation of fast myosin heavy chain isoforms. Exp. Cell Res..

[37] Chemello F., Grespi F., Zulian A., Cancellara P., Hebert-Chatelain E., Martini P., Bean C., Alessio E., Buson L., Bazzega M. (2019). Transcriptomic Analysis of Single Isolated Myofibers Identifies miR-27a-3p and miR-142-3p as Regulators of Metabolism in Skeletal Muscle. Cell Rep..

[38] Lena A.M., Mancini M., Rivetti di Val Cervo P., Saintigny G., Mahe C., Melino G., Candi E. (2012). MicroRNA-191 triggers keratinocytes senescence by SATB1 and CDK6 downregulation. Biochem. Biophys. Res. Commun..

[39] Boon R.A., Seeger T., Heydt S., Fischer A., Hergenreider E., Horrevoets A.J., Vinciguerra M., Rosenthal N., Sciacca S., Pilato M. (2011). MicroRNA-29 in aortic dilation: Implications for aneurysm formation. Circ. Res..

[40] Sun J., Li M., Li Z., Xue J., Lan X., Zhang C., Lei C., Chen H. (2013). Identification and profiling of conserved and novel microRNAs from Chinese Qinchuan bovine longissimus thoracis. BMC Genom..

[41] Sheng X., Song X., Yu Y., Niu L., Li S., Li H., Wei C., Liu T., Zhang L., Du L. (2011). Characterization of microRNAs from sheep (Ovis aries) using computational and experimental analyses. Mol. Biol. Rep..

[42] Schiaffino S. (2018). Muscle fiber type diversity revealed by anti-myosin heavy chain antibodies. FEBS J..

[43] Ahn J.S., Kim D.H., Park H.B., Han S.H., Hwang S., Cho I.C., Lee J.W. (2018). Ectopic Overexpression of Porcine Myh1 Increased in Slow Muscle Fibers and Enhanced Endurance Exercise in Transgenic Mice. Int. J. Mol. Sci..

[44] Dugdale H.F., Hughes D.C., Allan R., Deane C.S., Coxon C.R., Morton J.P., Stewart C.E., Sharples A.P. (2018). The role of resveratrol on skeletal muscle cell differentiation and myotube hypertrophy during glucose restriction. Mol. Cell Biochem..

[45] Meredith C., Herrmann R., Parry C., Liyanage K., Dye D.E., Durling H.J., Duff R.M., Beckman K., de Visser M., van der Graaff M.M. (2004). Mutations in the slow skeletal muscle fiber myosin heavy chain gene (MYH7) cause laing early-onset distal myopathy (MPD1). Am. J. Hum. Genet..

[46] Chen R., Jiang T., Lei S., She Y., Shi H., Zhou S., Ou J., Liu Y. (2018). Expression of circular RNAs during C2C12 myoblast differentiation and prediction of coding potential based on the number of open reading frames and N6-methyladenosine motifs. Cell Cycle.

[47] Burguiere A.C., Nord H., von Hofsten J. (2011). Alkali-like myosin light chain-1 (myl1) is an early marker for differentiating fast muscle cells in zebrafish. Dev. Dyn..

[48] Ravenscroft G., Zaharieva I.T., Bortolotti C.A., Lambrughi M., Pignataro M., Borsari M., Sewry C.A., Phadke R., Haliloglu G., Ong R. (2018). Bi-allelic mutations in MYL1 cause a severe congenital myopathy. Hum. Mol. Genet..

[49] Nawrotzki R., Fischman D.A., Mikawa T. (1995). Antisense suppression of skeletal muscle myosin light chain-1 biosynthesis impairs myofibrillogenesis in cultured myotubes. J. Muscle Res. Cell Motil..

[50] Cheung T.H., Quach N.L., Charville G.W., Liu L., Park L., Edalati A., Yoo B., Hoang P., Rando T.A. (2012). Maintenance of muscle stem-cell quiescence by microRNA-489. Nature.

[51] Kremneva E., Makkonen M.H., Skwarek-Maruszewska A., Gateva G., Michelot A., Dominguez R., Lappalainen P. (2014). Cofilin-2 controls actin filament length in muscle sarcomeres. Dev. Cell..

[52] Zhu H., Yang H., Zhao S., Zhang J., Liu D., Tian Y., Shen Z., Su Y. (2018). Role of the cofilin 2 gene in regulating the myosin heavy chain genes in mouse myoblast C2C12 cells. Int. J. Mol. Med..

[53] Del Coso J., Valero M., Salinero J.J., Lara B., Diaz G., Gallo-Salazar C., Ruiz-Vicente D., Areces F., Puente C., Carril J.C. (2017). ACTN3 genotype influences exercise-induced muscle damage during a marathon competition. Eur. J. Appl. Physiol..

[54] Musial A.D., Ropka-Molik K., Piorkowska K., Jaworska J., Stefaniuk-Szmukier M. (2019). ACTN3 genotype distribution across horses representing different utility types and breeds. Mol. Biol. Rep..

[55] Garton F.C., North K.N. (2016). The Effect of Heterozygosity for the ACTN3 Null Allele on Human Muscle Performance. Med. Sci. Sports Exerc..

[56] Staff P.G. (2015). Correction: Altered Ca2+ Kinetics Associated with alpha-Actinin-3 Deficiency May Explain Positive Selection for ACTN3 Null Allele in Human Evolution. PLoS Genet..

[57] Head S.I., Chan S., Houweling P.J., Quinlan K.G., Murphy R., Wagner S., Friedrich O., North K.N. (2015). Altered Ca2+ kinetics associated with alpha-actinin-3 deficiency may explain positive selection for ACTN3 null allele in human evolution. PLoS Genet..

[58] Lightfoot A.P., Cooper R.G. (2016). The role of myokines in muscle health and disease. Curr. Opin. Rheumatol..

[59] Lai J., Chen F., Chen J., Ruan G., He M., Chen C., Tang J., Wang D.W. (2017). Overexpression of decorin promoted angiogenesis in diabetic cardiomyopathy via IGF1R-AKT-VEGF signaling. Sci.Rep..

[60] Murata K., Morino K., Ida S., Ohashi N., Lemecha M., Park S.Y., Ishikado A., Kume S., Choi C.S., Sekine O. (2018). Lack of O-GlcNAcylation enhances exercise-dependent glucose utilization potentially through AMP-activated protein kinase activation in skeletal muscle. Biochem. Biophys. Res. Commun..

[61] Bullen J.W., Balsbaugh J.L., Chanda D., Shabanowitz J., Hunt D.F., Neumann D., Hart G.W. (2014). Cross-talk between two essential nutrient-sensitive enzymes: O-GlcNAc transferase (OGT) and AMP-activated protein kinase (AMPK). J. Biol. Chem..

[62] Zeng P., Han W., Li C., Li H., Zhu D., Zhang Y., Liu X. (2016). miR-378 attenuates muscle regeneration by delaying satellite cell activation and differentiation in mice. Acta Biochim. Biophys. Sin. (Shanghai).

[63] Carrano A.C., Liu Z., Dillin A., Hunter T. (2009). A conserved ubiquitination pathway determines longevity in response to diet restriction. Nature.

[64] Chen C.S., Bellier A., Kao C.Y., Yang Y.L., Chen H.D., Los F.C., Aroian R.V. (2010). WWP-1 is a novel modulator of the DAF-2 insulin-like signaling network involved in pore-forming toxin cellular defenses in Caenorhabditis elegans. PLoS ONE.

[65] Cao X., Xue L., Han L., Ma L., Chen T., Tong T. (2011). WW domain-containing E3 ubiquitin protein ligase 1 (WWP1) delays cellular senescence by promoting p27(Kip1) degradation in human diploid fibroblasts. J. Biol. Chem..

[66] Hao Q., Zhang Q., Li C., Wei S., Li Q., Song Y., Mi Y. (2017). A novel variant translocation (1;9)(p22;q34) resulting in a DEK/NUP214 fusion gene in a patient with acute myeloid leukemia: A case report. Oncol. Lett..

[67] Wise-Draper T.M., Allen H.V., Thobe M.N., Jones E.E., Habash K.B., Munger K., Wells S.I. (2005). The human DEK proto-oncogene is a senescence inhibitor and an upregulated target of high-risk human papillomavirus E7. J. Virol..

[68] Wise-Draper T.M., Morreale R.J., Morris T.A., Mintz-Cole R.A., Hoskins E.E., Balsitis S.J., Husseinzadeh N., Witte D.P., Wikenheiser-Brokamp K.A., Lambert P.F. (2009). DEK proto-oncogene expression interferes with the normal epithelial differentiation program. Am. J. Pathol..

[69] Wisniewski T., Newman K., Javitt N.B. (2013). Alzheimer’s disease: Brain desmosterol levels. J. Alzheimers Dis..

[70] Chesnokova V., Zonis S., Ben-Shlomo A., Wawrowsky K., Melmed S. (2010). Molecular mechanisms of pituitary adenoma senescence. Front. Horm. Res..

[71] Liu T., Krysiak K., Shirai C.L., Kim S., Shao J., Ndonwi M., Walter M.J. (2017). Knockdown of HSPA9 induces TP53-dependent apoptosis in human hematopoietic progenitor cells. PLoS ONE.

[72] Kim S., Myers L., Ravussin E., Cherry K.E., Jazwinski S.M. (2016). Single nucleotide polymorphisms linked to mitochondrial uncoupling protein genes UCP2 and UCP3 affect mitochondrial metabolism and healthy aging in female nonagenarians. Biogerontology.

[73] Dato S., Soerensen M., Montesanto A., Lagani V., Passarino G., Christensen K., Christiansen L. (2012). UCP3 polymorphisms, hand grip performance and survival at old age: Association analysis in two Danish middle aged and elderly cohorts. Mech. Ageing. Dev..

[74] Rose G., Crocco P., De Rango F., Montesanto A., Passarino G. (2011). Further support to the uncoupling-to-survive theory: The genetic variation of human UCP genes is associated with longevity. PLoS ONE.

[75] Patel A., Lee H.O., Jawerth L., Maharana S., Jahnel M., Hein M.Y., Stoynov S., Mahamid J., Saha S., Franzmann T.M. (2015). A Liquid-to-Solid Phase Transition of the ALS Protein FUS Accelerated by Disease Mutation. Cell.

[76] Lim J.S., Nguyen K.C., Nguyen C.T., Jang I.S., Han J.M., Fabian C., Lee S.E., Rhee J.H., Cho K.A. (2015). Flagellin-dependent TLR5/caveolin-1 as a promising immune activator in immunosenescence. Aging Cell.

[77] Khurts S., Masutomi K., Delgermaa L., Arai K., Oishi N., Mizuno H., Hayashi N., Hahn W.C., Murakami S. (2004). Nucleolin interacts with telomerase. J. Biol. Chem..

[78] Guo S., Zhang Z., Tong T. (2004). Cloning and characterization of cellular senescence-associated genes in human fibroblasts by suppression subtractive hybridization. Exp. Cell Res..

