# Supplementary figures and images for: Altered miRNA and mRNA Expression in Sika Deer Skeletal Muscle with Age
Source: Genes (Basel). 2020 Feb 6;11(2):172. doi: 10.3390/genes11020172 (PMC7073773; doi:10.3390/genes11020172)

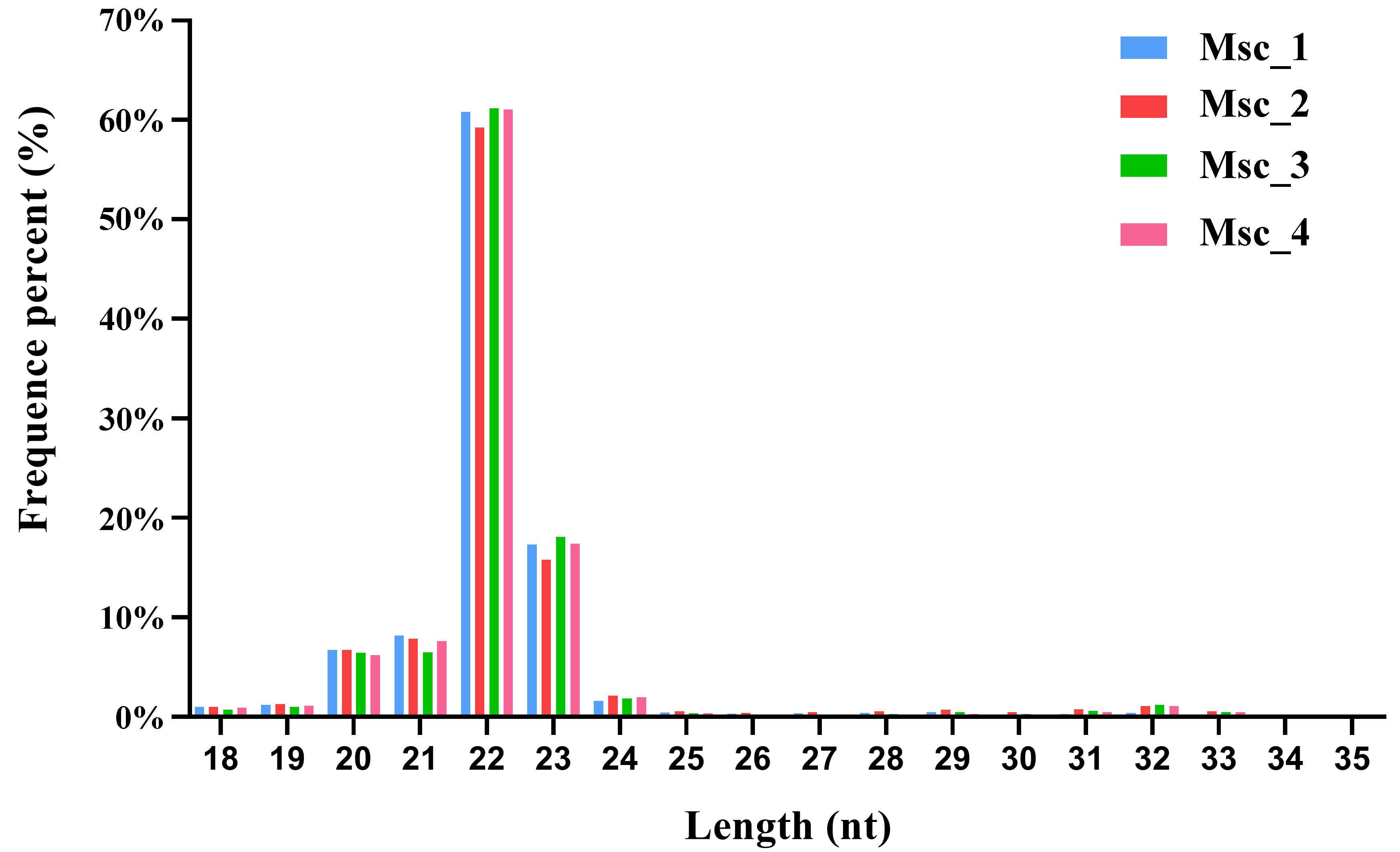

Supplement: Supplementary file 1 [file genes-11-00172-s001.zip › supplementary/Supplementary Materials/Figure S1.tif]

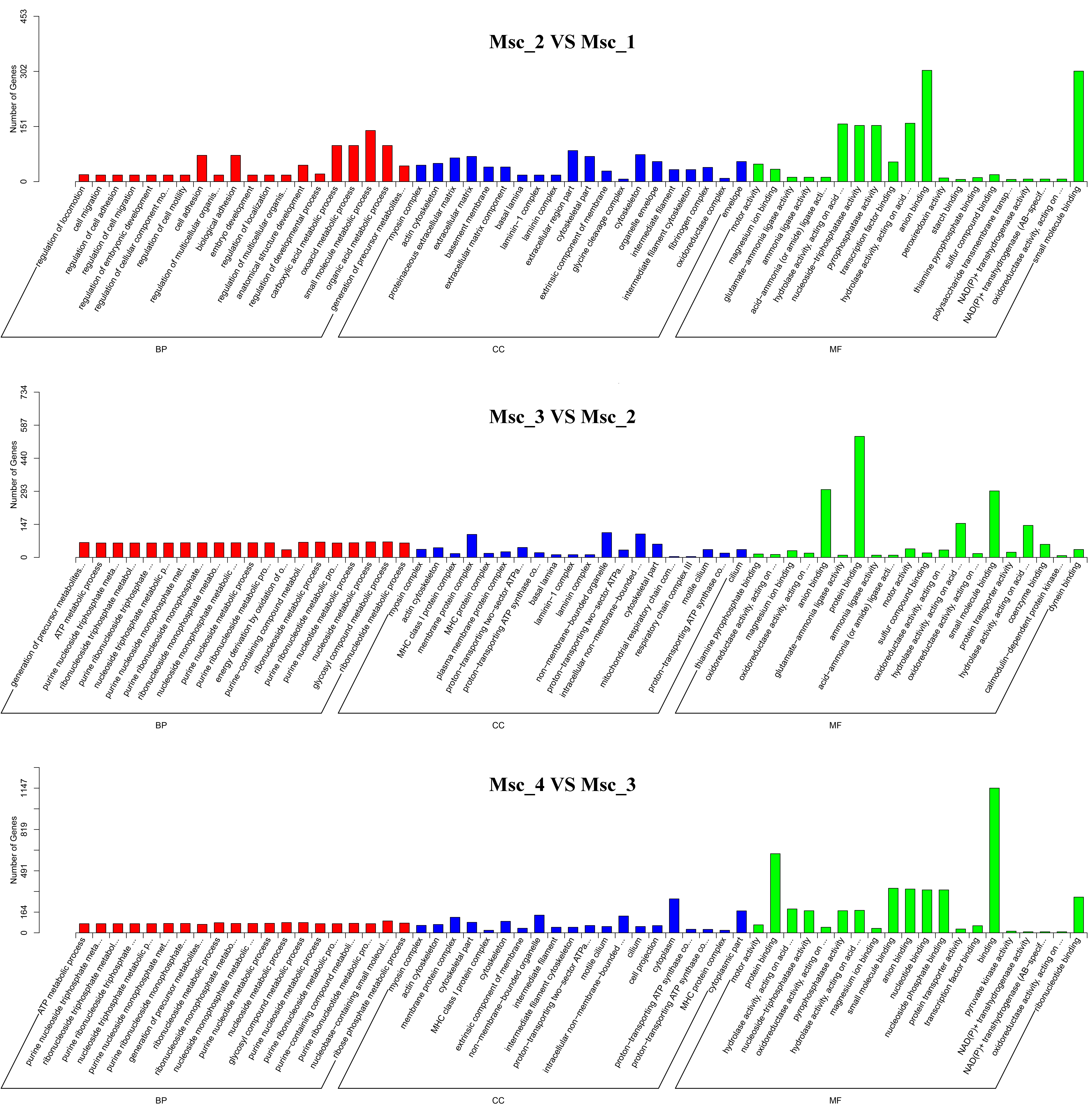

Supplement: Supplementary file 1 [file genes-11-00172-s001.zip › supplementary/Supplementary Materials/Figure S2.tif]

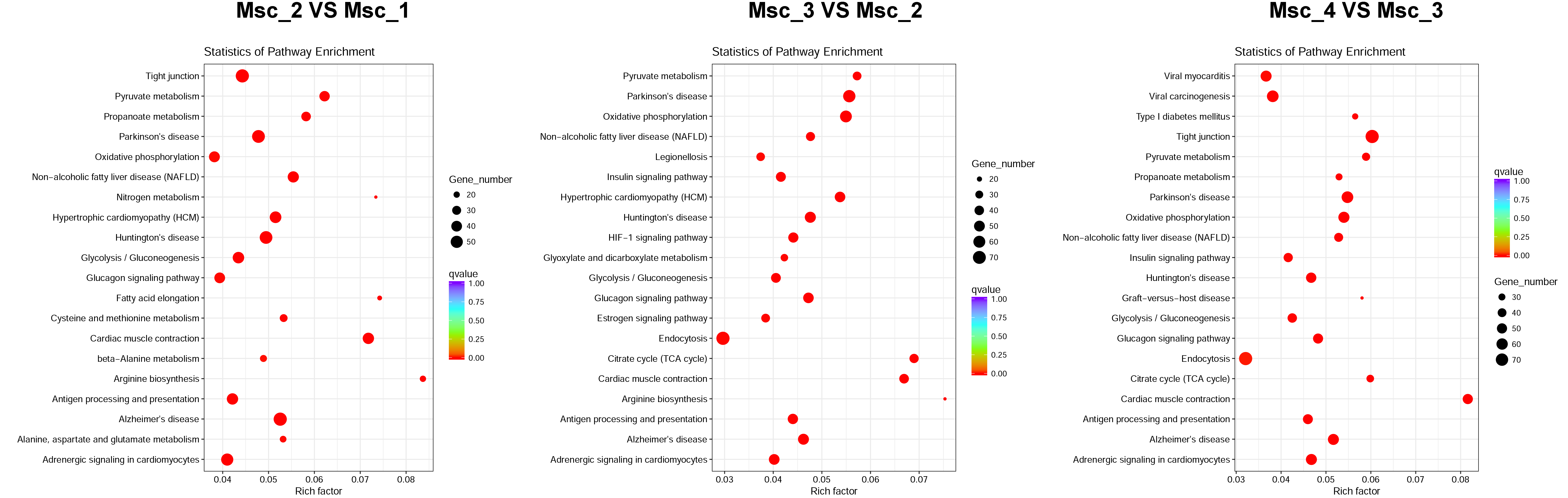

Supplement: Supplementary file 1 [file genes-11-00172-s001.zip › supplementary/Supplementary Materials/Figure S3.tif]

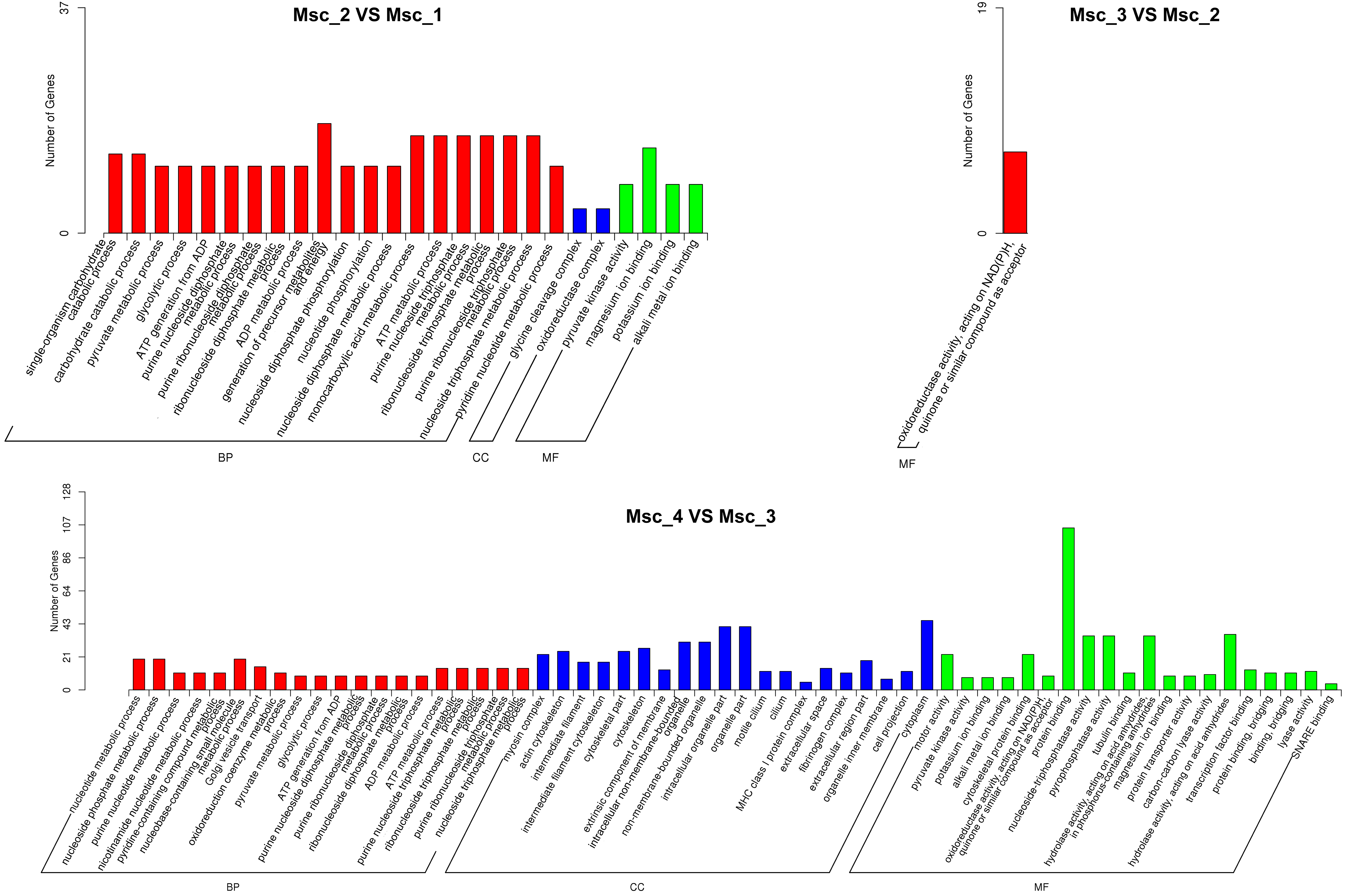

Supplement: Supplementary file 1 [file genes-11-00172-s001.zip › supplementary/Supplementary Materials/Figure S4.tif]

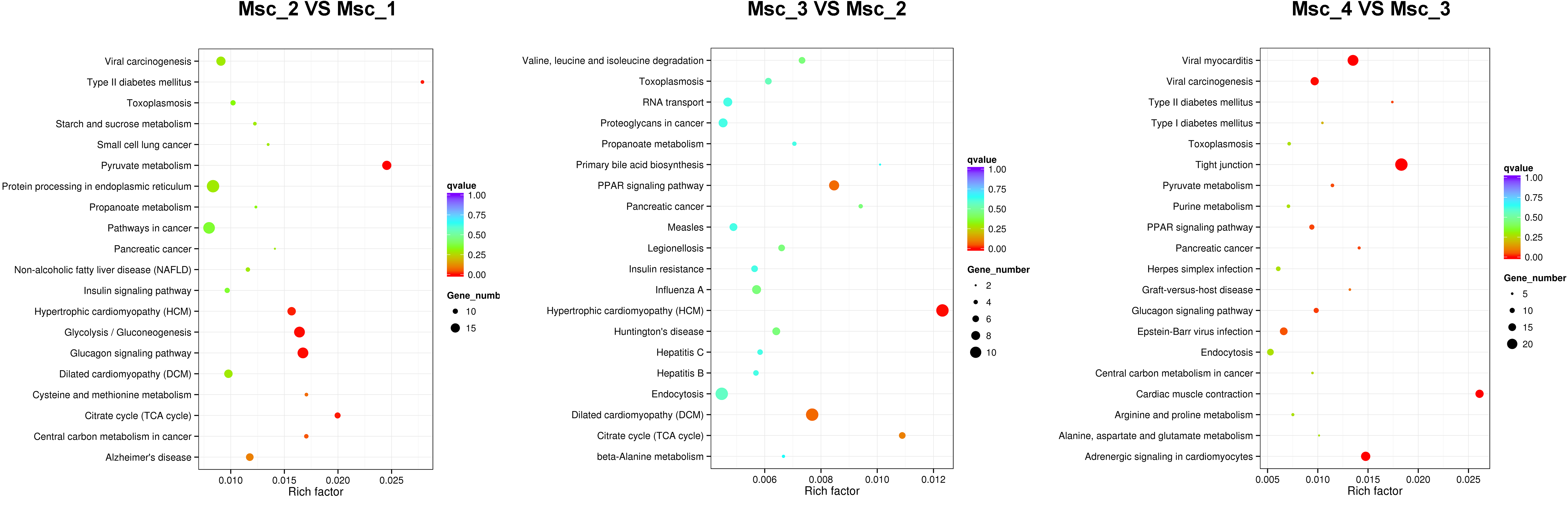

Supplement: Supplementary file 1 [file genes-11-00172-s001.zip › supplementary/Supplementary Materials/Figure S5.tif]
